# Selections and Abstracts

**Published:** 1906-04

**Authors:** 


### SELECTIONS AND ABSTRACTS.

### NUTRITION OR FATE?

By GEORGE B. H. SWAYZE, M.D., Philadelphia, Pa.

"I wonder why consumption can't be cured like other diseases?" This question was seriously asked by a young lady of delicate type and nervous temperament—one who for months has had an obtrusive and suggestive cough—the other day while gazing at the likeness of a novelist and playwright of conspicuous ability, the late Robert Neilson Stephens, who about a month ago died of consumption in Bournemouth, England. For ten years he had made a valiant fight for life by redoubled literary work in resistance to physical capitulation to premature death. Meanwhile he had sought different climates; the conservative value of clean air and systematic ventilation had been early impressed on his attention; he had availed himself of a regulation course of treatment at the Linford Sanatorium for the treatment of consumption near Ringwood Forest, England, that he might learn, as he expressed it in a letter to the present writer, "learn how to live during the long process of curing myself;" he had found at Bournemouth a location where he could more comfortably breathe the air, whereby he was enabled to pursue the mental advantages of literary work as relief from proneness to dejection; thus for several years after he could not ascend the stairs to have his room on second floors he outflanked death by the defences of his resisting will and intellectual occupation, by full feeding and flush nutrition, until a finishing grippe ended the contest. He had long before discerned that he could not exist if he inhaled the rasping, smothering gases, the smoke and dust of our bustling American cities. Because of such conditions he was driven from New York City one winter to the soft air of the Bermudas pending the reading of proof of one of his books. While many another would have succumbed in one year, this young man of iron resolve, after the severe initial stage or break, by persistently holding to the sanitary helm of daily and nightly ventilation, the importance of which had been pressed upon his intelligent observance, succeeded in making name and fame for himself among a hundred thousand readers in the interval of suffering before silenced by the relentless hand of doom.

In the broadening view of the sensible movement to reconstruct our tuberculosis theology, by allowing a beneficent Creator a free grace chance to help consumptives by natural laws of life—in lieu of conventionalized serum heterodoxy—the lesson of the distinguished example I here refer to is of deep practical importance to all doctors of medicine—especially to those who have inadvertently joined faith with the rubric of indefinite drugs, indigestible oils, closely warmed houses, and the flummery of tuberculin inoculations which hasten the fatal climax that so appallingly swells the records of the great white plague.

Young Stephens had belonged to my clientele in his earlier life. His father, Professor Stephens, had died of consumption in his country town in Pennsylvania. His brilliant mother had died of malignant cancer of the breast in Philadelphia — after a futile operation by the eminent late Professor Agnew. Nearly ten years prior to his death, young Stephens filled an important position that required much night travel from city to city in dramatic interests, compelling him to the makeshift of stuffy sleeping-cars behind gassing locomotives which wore severely on his by no means rugged vitality, and culminated in a severe influenza. He was then finishing his first drama, "An Enemy to the King," so ably presented to admiring throngs by actor Sothern the ensuing season. On visiting my client, I urgently explained that if he would protect his shaken lungs from permanent disease he would need resign his exhausting steam car travel contract, and seek the relief of more open air occupation. To recover from that attack of influenza he sought the sea air of Atlantic City as early as practicable. Once better, the remunerative venture was continued until an attack of acute pneumonia nearly cost his life. Impaired lung texture ensued, and the seal of consumption began to stamp its signals of disintegration. He relinquished travel, except in pursuit of sanitary breathing-air. Many a limited area evidently healed over with fibrous tissue. His devoted wife was his admirable nurse and care-taker—his unfaltering cheer-maker and inspiration. Thus with courage unimpaired, between the initial scourge and the final collapse, fighting for continuance of productive life day by day for nearly nine years, this invalid wrote seven dramas besides a series of nine popular novels, published by L. C. Page & Co., of Boston.

As medical men we are doubtless thankful for professional encouragement to be drawn from the lesson of one who so long surmounted the defalcations of tuberculous disease by the sunshine of a cheery helpmeet, by the beneficent ministry of daily and nightly sanitary breathing-air systematically maintained, and by the well-supported nutrition most faithfully promoted till he had successfully performed so great an amount of work for his post-mortem inheritance. Let us pursue the paramount study. Oxygenation is the stimulus and nutrition, is the leverage of life. Without either there is no life. The quality of the oxygenation of the blood corpuscles and tissues determines the quality of the nutrition and vitality or health of every individual in the world. The moment that we touch the quality of the blood we press the button on the quality of the nutrition of the body, all its cells and tissues, the texture of its organs, the integrity and the perfections—or the failures—of its functions, the proneness to or the exemptions from disabling disease. The unity of disease consists in the disability of vital functions, let the variations of physical expression be what they may. All disease is a deflection from the normal. The blood is the feeder and builder of every cell in the body. If the blood lack the qualities that sustain healthful physical integrity, disordered functions follow as naturally as shade is the reverse of light. Hence the recuperative agency of sanitary oxygenation of the blood gauges with nature's exactness the degree or quality of recuperative agency of nutrition.

No man cures disease, or heals wounds and sores, except through the process of nutrition. Nutrition is the consummation of life itself. The question reverts: — which impairs normal nutrition, plants a fundamental impediment to health—lays the foundation for disease in some organ or function. Since the body is material, normal nutrition is won by normal oxygenation only, and thereby the body is regenerated from its diseases. In order to heal, there must be a sufficient supply of healing material. Poor, anæmic, perverted quality of circulating blood means poor, anæmic, perverted quality of nutrition, of construction, and of function. There can be little mystery about causes of health and causes of most disease—even of consumption. The ground-floor problem is as simple—the philosophy as logical—as the mathematical principles of addition and subtraction. Excluding accidents—maintaining the physiological normal, restoring the disordered conditions back to the physiological normal is our precise task. The assistance of repairative nature is the master touch of all our successes. Unwise dosing with drugs that encumber and handicap nature is thrusting into the striving system the adverse weapons of hindrance and defeat. Medicines must be floated by the blood current—must be appropriated mainly by the reconstructive mechanism of nutrition. Medicines are comparatively like crutches, to assist the patient to hobble across the line of disability caused by sick blood and perverted nutrition.

Every tumor, every cancer, every neurasthenia, every tuberculosis proclaim the burrow and graft of perverted nutrition. Every asthma, croup, diphtheria, evince the grasp of poisoned deoxidized breathing-air, with the resulting perverted nutrition whereby the penalty in suffering must bear its cross. What we call diphtheria amounts to an ordinary brush of sore throat when the blood and nutrition are in sound or healthful condition. But the local scathing by poisonous elements in the breathing-air deepens into real seriousness when the blood and nutrition are depraved together. Malignant diphtheria means rot in the blood and tissues, else the healing would be relatively spontaneous and sure. The innocent bacillus found there?—blebbed into form and vision with the natural fermentation of physical decomposition?—how soon it vanishes from transient demonstration with a bit of healing! It is impossible for any bacillus to inaugurate disease, either in the throat or the lungs, if the blood be preserved in normal condition by healthful oxygenation. It is not the stunt of inhaling stifling fumigations that arrests either diphtheria or consumption—it is improved nutrition that regenerates the body from its diseases. Considering the natural simplicity of the problem, a dozen or a score of well chosen therapeutic remedials besides nourishments will carry a practitioner safely through a year's practice—allowing for a few special exceptions—while the inventive manufacturers are multiplying and flooding their markets by hundreds of confusing variations in drugs for the rounds of professional experiments and sales. I pity the doctors who pliantly skip from a really good thing to the next and the next pharmaceutical experiment—come day, go day—which way the bug hops!

We dare not—at least I do not—waver from the radical fact that perverted or disordered nutrition constitutes the warp and the woof of most disease. Breathing-air circumstance is the weaver. Omitting smallpox and a few contagion addenda, and also the accident of scalds and burns, even the condition for cure in these lesions depends on the repairative qualities of the blood and nutrition. And since consumption, which is not contagious, is caused by vice in the breathing-air habitually inhaled and by the misfortune of malnutrition, a reformation in these radical conditions afford the only promise of relief and cure when cure is yet possible. The strenuous fad of outdoor existence, eat and sleep, summer and winter, will presently be so overdone that it will lose prestige. Too many of those who are reported markedly benefited by the tent regime early relapse after returning to their accustomed zones of life—die and are buried just the same. It's queer—how over-strenuous doctors become when they get started that way? All in all the human family are a class of civilized humans—and not Norwegian bears! However, by the habit of years, the first thing that appeals to my attention on entering a house where illness has developed, is to glance after the manner of heating and ventilating; to see if there are any gas stoves or coal oil stoves to perjure the house air; to discover if stealthy fuel gases from heaters or stoves invade the rooms; to observe whether or not the bed or the crib is too close by the drift of the hot-air register; to see if the rooms are closed and even the shades drawn against the relief of refreshing outdoor air;—because I am alertly aware that there always exists a local preparatory condition from which has been more or less directly developed the affliction to which I have been called. At the outset it is my resistless desire to arrest the injurious influence, and to marshal every natural advantage to my assistance, rather than depend on the less certain virtues of my drugs unaided. In eight cases out of ten my treatment begins by opening a window to improve the repressed ventilation and the chances of my patient. For upon the quality of the oxygenation depends the quality of the patient's blood—for the blood of the body is the life of the body—that determines the status of constitution that is yielding to the grievance of abnormal conditions. I must talk from the heart out. I could win no rest of spirit if I went away from my patient and left behind me masked dangers that could muffle the sick one with stealthy and vital depressants during the interval of my absence.

The writer is not presenting the subject thus earnestly as a formal didactic affirmation. He realizes that he may be speaking parting messages to the world. There are great but overlooked truths in the care of the sick that eminently merit the "verily, verily," because they are the roots from which may be grown the daily salvation of the invalid. It has become the vogue to feed the consumptive to the limit of capacity. But the prestige of normal nutrition resides in the normal oxidation of nutritives while in the circulation, also in their subsequent elimination to make way for fresh supply. Quality and completeness gauge the grade of repair and integrity of functions. Foods can not be properly utilized unless normally elaborated by the chemistry of nature. The old needs to be resolved and dispersed to make way for the new, else nutrition and foods depreciate together.

It has been authoritatively stated that the adult person annually requires an average of about six hundred and fifty pounds of semi-solid and an equal quantity of fluid matter, and seven hundred pounds of oxygen to maintain an organism in a normal state. But this thirteen hundred pounds of gross material would seriously fail in the needful functions of normal nutrition, were it not for the transforming virtues of the seven hundred pounds of oxygen needed to render oxygenation sufficient to prevent deficient and perverted nutrition. Since oxygen is the vital transformer of the agents of nutrition, and a normal ratio of oxygen is furnished by pure air only, or its approximate, it must be clearly recognized that if breathing-air is deficient in oxygen and is impregnated with poisonous gases of any kind, there results defective and perverted or malnutrition—always an inflowing tide to some form of disease. All the "starvation neuroses" mean defective nutrition through lack of oxygenation of the blood and normal denutrition of the body because of pest-haunted breathing-air. Under the search-light of daily and nightly environment we may soon discern that not only the neuroses of children and adults, but many other common afflictions, including throat disorders, grippes, pneumonias and consumption are the physical results of inhaling poisonous elements prevailing in the local breathing-air of susceptible individuals by careless habits of managing the combustion of our ordinary fuels and illuminants—these aggravated by lack of ventilation, by close heat, house dust, smoke, sewerage and rebreathed human decay.

There is recognized often annoying debility and irritability of the system. We are wont to say that he or she is out of temper or not feeling well. But there is poisonous dead waste present in the system—accentuated by lack of normal nutrition. The feeding end of the dilemma is embarrassed by lack of the oxygenation and adjusting relations of foods. The organism is hampered with toxic debris. If we send our child out to regular exercise in the fresh air, how soon has its oxygen promoted enjoyable appetite, restored nutritive functions and revived spirits! But what of our house hermits?—our recluse shut-ins?—our devotees to shops or offices?—to unfriendly gas stoves and home cares?—to needy needles and sewing machine toils?—to the exhausting night lamp tax or chest-flattening desk?—to sweat-shop tasks?—to stuffy railroad jaunts?—to foundry and factory smudge?—to overcrowded sleeping-rooms, where purifying and vivifying ozone must forego its beneficent ministry?—lungs! lungs—all the wear on the lungs!—what chance has repressed mortal nature to normal elimination of decaysome and irritating waste of wornout material that corrupts the main channels to decent health? The abused lungs, consigned to the double task of taking into their cells degenerate air with every breath, and throwing off the corpse of dead carbonaceous matter from the venous blood with every breath, who wonders at their breakdown from the initial bronchial cough to the finale of fatal consumption? Let us study out the problem:—with the rasp and grind of continuous unhealthy environment, can sensitive lungs be expected to maintain a sufficiently defensive nutrition of their own to withstand a wear more scathing and degenerative than cast iron or steel texture could endure? Will not lung surface, cells and texture yield to death under such common wear and surrender to the characteristic disintegrations of tuberculosis? Or have we need of first ordering into action a supposed brigand bacillus to ravish the field of aeration—to degrade the service of nutrition and denutrition—to pillage and deplete the store of human vitality? Fudge! The bacillus is but the afterclap adjunct in the situation—in itself of no account.

This paper deals with the start of consumption. Heredity is not to blame. Heredity is outrun in the first two years of the child life in case the mother has nursed the infant from her own breast. Other nutrition displaces every element imparted by the mother. Environment is the master in the situation. After environment there is nothing to question. What may be accomplished in a case after initial lesions, is authoritatively illustrated by the remarkable history of the struggle and courage of author Stephens to prolong useful life by diligent employment of fresh air hygiene in his rooms—by the sustaining flush of extra nourishments—by his persistent diversion from self with daily intellectual occupation that had money values and was in harmony with his tastes. It was a great and unusual achievement for a dying man. The dogma about bacilli worming holes into his lungs was never given a thought—he told me that he didn't care a rap about such silly talk, the doctrine was as luny as moonshine for lunch—and he had the temerity to continue to live and to work till the accident of another grippe brought the finale.

1726 North Twenty-second street.—*Medical Times, April, 1906.*

---

### The Preliminary Training of a Surgeon; a Word to Young Men.

By A. C. Bernays, M.D., St. Louis, Mo.

The problem of the evolution, education or, if you please, of the making of a surgeon is one that has always greatly interested the profession. I am one of those who agree with the view of Osler that the creative, pathfinding or epoch-making ideas come to all men before they are forty years of age, although some may not publish or elaborate them until they are older. I am therefore of the opinion that the making or education of a surgeon should begin early. I am convinced that a silk purse can not be made out of a sow's ear and I know that the gold must be in the ore, or no process of refinement can possibly evolve or extract it. I know also, however, that young men with very limited training often outstrip some of their fellows who have had great scholastic advantages. There must be something in these untrained men which schools and lessons and other educational factors could not supply. Exactly what this something may be is very hard to say. My opinion, in a few words, is that the aspirant must belong to that type of individual whom Mr. Hadley, the president of Yale College, has called a *fact-seeker.* The young person, male or female, must be one of those whose hands, as well as whose brain, have been educated. I may be better understood if I say, he must be one of those who has learned to use all his five senses. He must be able to arrive at correct conclusions by accurate observation, that is, by the use of his senses. We have recognized that the highest function of a surgeon is to make a diagnosis. The ability to treat disease is entirely dependent on the recognition of the pathological condition. The operator will always rank just next to the diagnostician but can never be considered to stand higher than the diagnostician. It is much to be striven for that both functions be combined in one man, but experience has proven that two can be relied upon better than one alone. It is wise to have two kinds of talent in all obscure cases. Obscure and dangerous cases tend to make us modest, so that the suggestion of calling consultants is more readily accepted than in simple clear cases in which consultations are not only useless but expensive and harmful.

Besides being a man of educated, trained senses, I think the young person taking up the study of surgery should be self-reliant, full of sympathy with mankind, and last, but not least, should have a love for the acquisition of scientific truths. In addition to these basic traits, every quality of mind which goes to make an honorable, cultured gentleman is desirable.

It seems to me that an education in a scientific school, such as Sheffield School, or the Massachusetts Institute of Technology, would fit a young man better for the life work of a surgeon than a college course leading to the A.B. degree. At institutions like those mentioned, physics and chemistry are taught, without which an understanding of biology and physiology is simply impossible.

The education of young gentlemen in America will lead to the making of a better class of surgeons than an early education in Continental Europe or England, in the fashion prevailing at the present day. At country parties in Europe I have seen young gentlemen absolutely helpless in cases of accident. They could neither saddle a horse nor unhitch a team in an emergency, although they were stylish riders and drivers. You must know that the European gentleman has his sons and daughters trained in riding, driving, swimming, shooting, etc., by a teacher from whom they receive lessons in these arts. Our American boy learns these accomplishments by hard experience, which, after all, is the best school. In most cases, we learn to shoot, by disobeying our parents, and so it is with other accomplishments of a sporting and athletic character. Our boys are therefore more practical and useful in an emergency and become more resourceful in a surgical operation requiring manual dexterity and a cool head.

Our young man is now ready to enter a medical school and the first year ought to be devoted to anatomy, physiology, embryology and organic chemistry. In the second year he begins pathology and continues histology. During these two years, nearly all his time should be spent in the dissecting room and the laboratories. Having taught anatomy for eighteen years in various medical colleges, I may be permitted a few remarks about the study of this subject. Medical men have always looked upon it as an auxiliary, because upon it is based the art of surgery. Until recent years, our knowledge of anatomy depended upon the dissection of human and other animals. When we look back over the field we are more and more astounded at the enormous number of facts we have learned about the structure of the human and other organized bodies by dissection. Until recently, our knowledge rested on microscopical and macroscopical dissection. Recently the modern science of embryology has given us a more stable and scientific basis for our anatomical work. Suppose you are desirous of investigating the structure or construction of a complicated machine, for instance, a man-o'-war. It is not at once clear to you that you can understand its structure much better if you watch the building of such an engine of war, than if you tear one to pieces or dissect one? There can be no doubt about the best method to pursue. Watching the development and construction to its completion of a complicated machine will at once give you an understanding of all its parts and functions, whereas the mere dissection or taking apart of the machine will not give you this understanding, although it will acquaint you with the parts.

Remember that anatomy which is learned by memory is easily forgotten and is often useless, but anatomy which you have grasped by your reason and understanding will last forever. For example, let us take a question in anatomy: Why does the recurrent laryngeal nerve dip down into the chest forming a long loop, around the arch of the aorta on the left and around the subclavian artery on the right, in order to reach its end in the muscles of the larynx which form the voice? A man who has studied embryology at once answers the to him self-evident question, and an anatomist whose knowledge rests on dissection only, can not answer it at all. Let me therefore caution you again not to depend on anatomy which you have committed to memory, but learn it by the exercise of understanding and of reason. Learn to understand the structure of the body, on which depends your ability to do surgery, by studying its development from the egg stage to completion. Your knowledge of anatomy and of physiology must not depend upon what you read in books; it must be founded on observations made by yourself, by the use of your senses, and by the use of instruments of precision. You can have no scientific knowledge as long as you depend upon the authority of books and lectures. Science abhors authority. It requires demonstration of facts, it believes nothing upon any one's authority.

But very few hours can be given to didactic lectures. Actual work in the laboratories, repeating the fundamental experiments of biology, is the student's main occupation and this he may vary as much as his inclination or his genius will prompt. It is not at all excluded that the young student may make important modifications of existing methods; indeed, he may make discoveries. He will probably find many facts that are not mentioned in the text-books. These he will at first believe to be discoveries, because he will expect too much from his text-book. But before long, if his work continues on proper lines—and by that I mean continued laboratory study with the aid of a teacher, who is an investigator or researcher—he will soon find out that a text-book is a poor but necessary assistant and guide. He will soon find the text-book a most incomplete and unreliable authority in scientific experiment and research.

In the second two years the student takes up the practical departments, and nearly all his time will be spent in practical courses and in clinics. Again he will consult text-books, but he will have learned that observation at the bedside is his most attractive and most profitable method of study. Attending didactic lectures on surgery and medicine he will find a waste of time, and the brighter students will "cut" these didactic lectures because they learn more at home from a good text-book than from a lecturer. The reason for this may be illustrated by an example. I know a doctor who is a bright, glib talker, but he is a very poor pathologist and only an average surgeon. Nevertheless, he became Professor of Surgery, and I believe still holds that position. He teaches the principles of surgery and I am told he does it to the liking of his hearers. No one will pretend that his lectures will compare at all with Senn's beautiful and clear chapters, but all know that the lectures are simply taken from Senn's book. They are a weak, degenerate and incomplete rehearsal of the text-book. No further argument is needed. His students would gain by studying the text-book and "cutting" the lectures. During the last years, the student will do best to spend all his time in clinical courses. Hospital clinics, obstetrical, psychiatric, orthopedic, in fact all varieties, must daily engage most of his working hours. Only a few hours must be reserved for the reviewing of notes by the aid of text-books.

The student will now be graduated and then comes the most important step in the making of a surgeon. He must become attached to the staff of a hospital or become assistant to a surgeon who has hospital connections, so that he may take part in the daily exercise of the science and art of surgery. Exactly how many years should be spent thus, I can not say. One, two or three years should suffice to turn out a surgeon who is capable and ready to offer his services to the public with a good prospect of making a useful and a successful diagnostician and operator.

Before closing this short memorandum on the making or evolution of a surgeon, let me add a few more reflections. I do not believe that any one can become a really good surgeon who does not think of himself that he can perform a resection of the pylorus, a resection of the three branches of the trigeminus or the extirpation of the Gasserian ganglion nearly, or quite as well, as any other surgeon in the country.

Neither do I believe greatness can be achieved in surgery by any one who does not, during the first five years of his practice, remove some malignant growth from the face or neck of an unfortunate fellow which have been pronounced inoperable by some old and prominent surgeons. It follows that I am of the opinion that surgical technic will continue to improve and that surgery must not be thought a finished art.

Finally, let me say that a great surgeon can not be recognized by the public. They have no way of judging between a good doctor and a quack, or between a real surgeon and a mere pretender. The cardinal way of judging of a surgeon's work is to see it done and to be allowed to see the final results of the operations. A great surgeon will therefore always be willing to invite colleagues, young ones as well as prominent ones, to witness his operations, to watch his after-treatment and to study his results. An infallible sign of a great surgeon is his willingness not only to show results, but to teach and demonstrate his methods to students and colleagues from any quarter of the globe.

The acquisition of scientific truth for the benefit of mankind, is made as easy and free as the air we breathe, by the great universities. Our rich men, by endowing these institutions, are doing great deeds for their fellow men of this and of future generations. Great surgeons owe it to mankind to make the *art of surgery* as accessible and free as is the science.

---

### BACKACHE IN WOMEN AND ITS TREATMENT.

By WILLIAM E. PARKE, M.D., Philadelphia, Pa.

There is a widespread popular belief that backache is due to kidney disease or womb disease. This belief is fostered by well-known patent medicine advertisements. That it is a very frequent symptom in women coming to a gynecologic dispensary service is familiar to all engaged in that sort of practice. I have looked over the record of one thousand consecutive dispensary cases, and find this symptom especially noted in 252 instances, or 25 per cent. My offhand impression was that not less than 75 per cent. of this class of patients complained of this symptom. I am confident that the former figure is an underestimate—due, on the one hand, to incomplete notes, the patients themselves not mentioning this symptom because others were more unusual or more urgent; and on the other to the fact that many of these women were not sick, but applied simply for diagnosis as to pregnancy.

The seat of the pain is the lumbar, the sacral, or the coccygeal region. Its cause is not always easy to determine. The lumbar pain or ache is usually an expression of some general disorder, whereas the pain which is distinctly circumscribed and limited to the sacral or coccygeal region, is often directly or indirectly due to disorder of the pelvic viscera, such as lacerations, displacements, inflammation, or tumors. The most frequent cause of backache is the neurasthenic state—a very common condition in modern strenuous city life. It is an evidence of nerve tire, of bodily and mental fatigue. The neurasthenia itself may, however, have for its underlying cause some pathological condition of the pelvic organs, such as those mentioned above, which give rise to changes in circulation, lack of support, etc. There is thus indirectly a relation between the pathology of the pelvis and the backache.

The history of Mrs. M. S. is quite a typical one. A hard-working woman. She enjoyed good health until her first baby was born, at which time she sustained a tear of the cervix and perineum, which was not repaired. She never felt so well after the baby was born as before. After an interval of two years a second baby was born without apparent added lesion in the birth canal, but there probably resulted still further weakening of the pelvic support. Now she complained of constant backache, bearing-down sensation, and lack of sense of support. She dragged herself around, did all her work, but life was a burden. Examination at this time showed her to have a torn and relaxed perineum, a torn cervix, a somewhat enlarged and retroverted uterus, with some tenderness of the appendages. Now the backache, which did not appear in this instance until after the second baby was born, was not caused directly by the lacerations, which occurred at the first labor, but is an expression, I take it, of nerve tire or neurasthenia, in which the lacerations and displacements of the pelvic viscera are a contributing cause. Perhaps the backward displacement did not occur until after the second labor, and the backache was coincident with the displacement. There is of course more decided impediment to the circulation resulting from the sagging down of a heavy uterus and its final turning upside down, causing torsion of the broad ligaments, than from laceration without marked displacement. It is precisely in these cases that we find the most aggravating backache. Lacerations and displacements do, however, occur without backache. I have found forty cases of retroversion in this series in which it did not occur, or was so unimportant as not to be noted. These are the cases in which the uterus is small and the pelvic congestion is trifling. There was found in this series a much larger percentage of retrodisplacements with backache than without, the ratio being 194 to 40. While dwelling especially on this symptom I do not mean to imply that it stands out by itself alone. It is when due to pelvic disorder quite uniformly associated with pain in the left ovarian region and tenderness on pressure, and often with pain on both sides or over the whole lower zone of the abdomen.

I have found in this inquiry forty-six patients complaining of backache who showed no considerable lesion of the pelvic organs. One was diagnosticated "hypoplasia of the genital organs," and it is probable that some had the kind of ovaries that are labeled cirrhotic, in which severe dysmenorrhea is so commonly present; many were constipated.

Backache is a very distressing symptom of acute toxemias such as smallpox, influenza, and tonsillitis, and the minor degrees of pain may very well be due to more chronic intoxications such as that from intestinal absorption, and perhaps imperfect metabolism.

Many women suffer with backache at their menstrual periods, and an excessive flow is quite constantly attended with backache. A congestion, whether active or passive, appears to give rise to backache.

Pain in the region of the coccyx is usually so acute and so circumscribed in area, and its localization susceptible of demonstration on motion, that one would expect it to be always due to a local disorder, and yet experience often shows it to be a pure neurosis. I once removed a coccyx of this sort, but the patient kept on having the pain. Pain at this point sometimes follows childbirth, and after some weeks entirely disappears. Under these circumstances there is reasonable ground to suspect an injury, but a hasty operation may be a needless one. Where a fracture or a dislocation enteriorly or posteriorly can be demonstrated, then an operation is required for its relief. When no lesion is demonstrable, an operation may be uncalled for, and is sometimes, as in the case above referred to, useless.

Many important diseases arising in the bony structure, the kidneys, the nervous apparatus, and the muscles are attended with pain in the back, and may sometimes have to be differentiated from pelvic disease. It is not my purpose, however, in this brief paper to include all the causes of pain in the back, but principally those that come to one's notice in a gynecologic practice.

The treatment of backache is involved in as much complexity as its cause, and often baffles one's best efforts, at least among the poor, who can not adopt the rest measures that are sometimes required.

In the case of lumbar pain where the cause is considered to be some toxic substance which is retained in the body, it is my plan to keep the bowels well cleared out, using for this purpose aloin tablets, cascara, or some saline interchangeably, with occasional doses of a mercurial. Sometimes colon irrigation is substituted, in which two quarts of saline solution is slowly passed into the bowel. In conjunction with either of these methods I direct that a definite amount of water be drunk each day—six to eight glasses, to which is added one teaspoonful of potassium bitartrate once, twice, or three times, according to the state of the bowels. This is with a view to increasing the secretion of urine.

Sometimes, loosely adopting the patient's ready-made diagnosis of rheumatism—which I have been the more ready to do in the absence of other tangible cause—I have given salol, salicylates, or the iodides, and with very indifferent success. Now and again such marked improvement has followed the administration of this class of drugs that I have been encouraged to repeat it in apparently similar cases, only to be utterly disappointed.

Basing my therapy on the assumption that many backaches are due to congestion, active or passive, I have given ergot in doses both large and small with a view to narrowing the caliber of the vessels. Sometimes I combine it with digitalis and strychnine. In those cases of backache accompanied by excessive uterine hemorrhage these drugs have a positive value. I am prone also to use the bromides with the double purpose of lessening the pelvic congestion and dulling the sensibility to pain. To a considerable number of these patients suffering from constipation, anemia, and enurasthenic symptoms, I give iron and other tonics in addition to correcting the constipation, which in the long run often yields gratifying results.

Whatever be the cause of backache, temporary relief is to be had by the use of counter-irritation in its various forms—capscine and belladonna plasters, liniments, sinapisms, cups, and blisters. I have used them all. They afford a ready and prompt means of allaying pain.

In that large class of backaching of patients in which neurasthenia in its milder forms plays so large a rôle, we are often not able to adopt the approved method of treating neurasthenia. As well might we "call spirits from the vasty deep" as to direct our poor patients to go to bed for six weeks and be given massage and electricity.

So much for the distinctly medical aspect of these cases. There remains a large proportion of patients who require for cure some surgical procedure.

Prophylaxis is, of course, in order here as elsewhere. Lacerations should be guarded against, and when occurring should be immediately repaired. Retrodisplacements are far more amenable to cure when treated promptly after labor than when allowed to become chronic. The backache and bearing down which result from sagging of the pelvic viscera and incomplete retroversion of the uterus are commonly relieved by the application of tampons and a properly adjusted pessary. More often they should be operated upon. This is more especially true of the hard-working poor patients, who can ill afford the time for an indefinite course of treatment, and are rarely permanently cured. Over and over again such patients, declining operation, make a few visits to the dispensary for local treatment, are relieved, and discontinue their visits, only to return after a few weeks or months with the same train of symptoms.

When the menstrual flow is so copious and prolonged each month that the loss exceeds the gain, curettage may be resorted to. This, however, is a mere expedient. The underlying cause should be sought, and sometimes it is an endometritis, sometimes an inflammation of the appendages, sometimes a tumor. Whatever be the cause, appropriate operative treatment should be resorted to. With the cause removed the patients will get well. Recovery from the frequently associated neurasthenic condition is promoted by the rest in bed, selected diet, and tonics.—*Therapeutic Gazette, February, 1906.*

---

### THE BITES OF INSECTS.

By ISADORE DYER, M.D., New Orleans, La.

In the ordinary divisions of diseases of the skin one group includes the animal parasitic diseases. In this division are embraced a variety of insects which are truly parasitic upon the human skin, either living in the skin and feeding on contiguous tissue, or else living in the appendages of the skin, the clothing, and feeding on the human blood. This group embraces the *Sarcoptes scabei,* the *Demodex folliculorum,* the three varieties of *Pediculus,* the sand flea (*Pulex penetrans*), and the *Pulex irritans,* or common flea; the guinea worm, *Cysticercus, Echinococcus; Leptus autumnalis,* or red-bug, the *Ixodes,* or tick; the *Cimex lectularius,* or bed-bug; the *Culicidæ,* or mosquitoes; *Simulia,* or midges; bees (*Apes melliferæ*), and wasps (*Vespidæ*).

Only here and there in the history of dermatology is any reference made to the characteristics of these, and then only in their relation to some other disease. The researches of Nutall, Manson, Laveran, and the numerous other experimenters with mosquitoes, have shown a direct relation to systemic infection. The domestic fly and the bed-bug have now for some years borne the burden of accusation in the responsibility for local and general infective conditions. Speculation has arisen at various times regarding the role other insects play in a variety of diseases. These have been overshadowed by the peculiar prominence which has attached to mosquito investigation.

The present paper is briefly directed at calling attention to the local evidences which present in each of the conditions for which insects are responsible; it is intended to attract attention to the early recognition of the lesion, or lesions, which each presents so that they may be recognized when seen.

It is curious but true that no two of the parasites and accidental insects which attack the skin show an identical lesion under these circumstances. Any area of attack may on its own merits be differentiated at a close examination.

We may divide the parasitic enemies of the skin into two general divisions; those which are common to the human skin, and those which are extraneous. In the first group belong scabies, the pediculus, demodex and filiaria. In the second group belong all the rest of those enumerated.

Scabies presents a vesicle at the point of its burrow; the pediculi are marked by papules or pustules from infection. The guinea worm forms a tumor which ulcerates.

Now all of the other parasites named above present as the evidence of their presence some sort of a wheal. In some instances the circumscribed edema is caused by the presence of the parasite in the skin itself, viz.: the tick, red-bug, cysticercus, etc. Others occasion the lesion either by their selection of the human skin for feeding purposes, *e. g.*, the flea, bed-bug, the mosquito, or the lesion is the result of a venom infection occasioned by an inimical incision by the proboscis or other instrument of the insect disturbed in its natural pursuits. In this last case we would include the bee, wasp, spider, etc.

A careful study of the clinical evidences of each of the above groups and their individual components would show that even then differences are to be noted.

The *Demodex folliculorum* is actively associated with acne and rosacea, and in its occupancy of the orifices and ducts of the fat glands occasions a papule, irregular in size.

*Ixodes,* or wood-ticks (Nat. order *Acarina*) bury their proboscis in the skin and determine a small wheal at the point of entrance; the body of the tick grows in size until the tick is thoroughly engorged, when it falls off.

The *Leptus autumnalis* is the familiar "red-bug" of this section; it is a six-legged larva of the species *trombididæ.* It finds its sites of selection on the legs, but may travel to the armpits, cruroscrotal region or over any part of the body. The lesion is either a small red papule, or a wheal, hard, rounded, and with the bug in the center. The insect drives its head into the skin and the intense itching resulting occasions the victim to scratch a central hole in the lesion in the endeavor to remove the head of the bug.

*Pulex penetrans,* "sand-flea," "chigoe," "jigger," is found in dry sandy soil in tropical and subtropical countries. The fecundated female burrows into the skin, usually of the feet, between the toes, but the insect may travel to other protected parts of the body. The lesions are vesicles, usually numerous, as the irritation is intense and the resulting scratching may occasion extensive dermatitis or even ulcerative processes.

*Pulex irritans,* or the common flea, friend of the dog, his usual host, or the hog, or the cat. The flea is a bloodsucking breed and bites either in search of food or for protection. The lesion is always small, the size of a half-pea—is a wheal and almost invariably occurs in pairs. No other insect bites twice as does the flea, with the two lesions arranged like dumb-bells.

*Cimex lectularius* is the euphonious name of the common bedbug, which is only too familiar. It is an accidental parasite on the human skin, sucking blood, or biting for defense. The puncture of the proboscis of the bed-bug carries with it some toxic element which is productive of intense inflammation, locally, and at times resulting in severe constitutional irritation, with fever or severer symptoms. The central puncture, often hemorrhagic, is surrounded by a halo of edema, fantastic in outline, classed as wheal, but varying from a simple reddened area to a gyrate lesion as large as the hand or even larger, with swelling out of all proportion to the evident exciting cause. The itching is intense and excoriations frequently mark the site of the edematous lesions.

Some *Flies* attack the human skin, usually nipping a small papule to the surface. As a rule the edema is not marked.

*Ants* biting the skin create small hemorrhagic papules with a burning sense of irritation rather than the itching associated with a deeper intoxication.

The *Spider* of ordinary varieties biting the skin usually does so with venemous instinct. The central point of the wound is always characterized by a small vesicle, surrounded further by a circle of edema. This circle of edema is as typical of the spider bite as is the central vesicle. At times, on sensitive areas, as the scrotum, a marked edema may additionally develop around the original lesion. This will depend upon the venomicity of the insect.

With such spiders as the tarantula, etc., attacking the exposed parts, and especially the hands, a rapid destructive lesion forms. It is initiated with a vesicle, which increases several times its original size, with a deep-seated edema associated. As soon as the acute evidences of the toxic infection is over, there are lines of definition of destruction and the area of original infection, the blister area, sloughs.

*Bees* bite only when disturbed and with a lincinating puncture, usually appearing as if double cut. The swelling is prompt and protuberant, rounded like a half egg in the skin, the lesion is usually reddened, but may become edematous wholly or in part.

*Wasps* and *Hornets* bite alike. The lesions are almost always on the hands and face and are multiple because these insects are ordinarily encountered near their nests, and there they seldom travel alone. It is unusual to see a single hornet or wasp attack the skin, and if such be so, it is only after continued provocation. The lesions of the wasp's and hornet's bites are small, highly inflamed and with a red center and edematous ereola. This last may merge into adjacent lesions, so as to give the impression of a suffused mass of edema with islands of red scattered all over the field.

*Mosquitoes* bite in two ways: If undisturbed the proboscis finds the tip of a papilla of the corium, sucks constantly until surfeited and then flies away; less carelessly the proboscis is driven into the skin without intelligence, it breaks between the papillary prominences, sucks away, draining blood from this area and as a result occasions an edema here.

The first method seldom annoys, while the second occasions intense itching and more readily disturbs the victim of the bite. In the first type of bite it is no unusual thing for a person to fall asleep and after half an hour or so to wake with a spotted face or hand. It is this small hemorrhagic, erectile papule, which dots the face like an eruption of measles, or rings the wrist or ankle with a double or triple line of red dots. The others are deep-seated, small, wheals, which itch, even recurrently, after the bite has been recorded.

There may be other insects which bite the skin, but as a rule the interest attaching to these is much more personal and individual than general. This paper is aimed at creating a more general observation of these things in order that with information arising from such direction the writer may deal exhaustively with the subject at a later date.

For the present it should serve some purpose to point out the differences in the evidences of the little enemies, that they may be duly recognized when seen.—*New Orleans Medical Journal.*

---

### SPINAL ANESTHESIA BY MAGNESIUM SULPHATE.

Haubold and Metzer (*Journal A. M. A.,* March 3), pursuing their work with magnesium sulphate as an anesthetic, report seven cases of spinal anesthesia, from which they deduce the conclusions that :

1. Magnesium sulphate in intraspinal injection proved to be as efficient an anesthetic in human beings as it was found to be in animals. In Case 7 a certain dose of magnesium sulphate produced local anesthesia in 45 minutes after intraspinal injection and about 3 hours later a deep general anesthesia set in which lasted for many hours and which would have permitted any operation on any part of the body. Although the dose used in this case was the largest we have employed in human beings, it was still less than one-half of the dose we have regularly used with monkeys with perfect impunity. The experience in the first two cases, however, has demonstrated that even such a small dose as 1 c.c. of the 25 per cent. solution to 25 pounds body weight is capable of bringing out complete anesthesia and paralysis of the lower part of the body, although not until two or three hours after the injection. The anesthesia and paralysis observed in these cases, appearing about two hours after the operation, could certainly not have been due to anything else but to the magnesium salt. Even the general anesthesia which set in late in these cases was undoubtedly, in the main, due to the intraspinal injection, since the small amount of chloroform which was employed in these cases could never produce such an after-effect. In Case 4 the patient apparently could have been operated on even at that early hour without chloroform. The absence of any noteworthy after-effects in this case was surely due to the washing of the spinal canal. The washing of the canal, although carried out at a comparatively late hour, proved to be beneficial also in the seventh case; the respirations increased and all the after-effects were perceptibly shortened. In Case 4 as well as in Case 6 there was a stage of analgesia which permitted the operation, although the tactile sense was not abolished.

Case 3 has shown that a dose of 1 c.c. of the solution of magnesium sulphate to thirty pounds body weight is insufficient to carry out an operation by its aid alone, although it apparently supported, in a measure, the effect of the chloroform.

In Case 5 even a dose of 1 c.c. of the solution to twenty pounds body weight had only a moderate anesthetizing effect. This patient was an alcoholic, fifty-seven years old, with markedly developed arteriosclerosis. Possibly alcoholism, as well as arteriosclerosis, interferes in some way with the anesthetizing effect of the magnesium salt. Possibly also there is an individual predisposition for or a resistance to the anesthetizing effect of magnesium. Our impression is that it affects younger individuals more readily than older ones.

2. It seems that the spinal injections caused a rise of temperature in all the cases. Although in the majority of the cases there were some other reasons for the appearance of fever, the setting in of the rise of temperature at about the same time and the similarity of the course of the fever in all cases made it probable that the intraspinal injection was essentially the cause. This is the more probable, since it was observed also in intraspinal injections of other substances. The course of the fever, however, was in all cases perfectly benign and disappeared after thirty-six or forty-eight hours. The pulse remained always of good quality.

Vomiting occurred in most of the cases. In Case 7, in which no chloroform was employed, vomiting occurred only twice and lightly, and only at an early stage. The frequent vomiting in Case 2 was probably not due to the intraspinal injections. It is noteworthy that in most of the cases the postoperative vomiting had only the character of regurgitation (no contraction of the abdominal muscles).

4. There was retention of urine, lasting between twenty-four to forty-eight hours, in nearly all the cases. The bowels also had to be assisted by enemata. The incontinence of feces of the first case was due to the involvement of the sphincter in the operation. We have to point out again that in Case 4, in which the spinal canal was washed immediately after the operation, there were practically no after-effects.

As far as seven cases permit any conclusions, the following statements may be made: Intraspinal injection of magnesium sulphate is capable of producing anesthesia, and, if carried out with caution, seems to be a safe anesthetic. The following plan would seem the best, at least for the present: To inject about 1 c.c. of a 25 per cent. solution of magnesium sulphate for every eighteen or twenty pounds body weight and wait about two hours. It is probable that by that time the analgesia will be sufficiently advanced to permit the performance of an operation on any part of the lower half of the body. A small dose of chloroform, however, should be administered to divert the patient's attention and to hasten and complete the anesthesia. Immediate washing of the spinal canal should follow the operation in all cases.

A more extensive experience will show whether this method could be used also as a means for general anesthesia for operations on the upper part of the body. It goes without saying that the solutions should be sterile, the salt chemically pure, and the patient handled with proper surgical care.

---

### ACUTE ARTICULAR RHEUMATISM.

Hargens recommends the following treatment:

There can be no jugglery in the treatment of this disease; the least digression from a few of the points mentioned means disaster; skepticism as to the existence of a successful treatment of acute articular rheumatism and the prevention of its complications is based on errors of diagnosis. By eliminating these errors of diagnosis and rigidly carrying out the treatment indicated, one will be eminently successful. Cases complicated by cardiac involvement are cases complicated by infection and one must recognize this element in treatment. I realize that in the treatment on which I insist there is one element very difficult to obtain, especially for those engaged in country practice. I refer to the bath-tub. Without the tub there will be more or less failure. To a certain extent this can be overcome by some improvised hot-air apparatus.

I have been profoundly impressed by the importance of two elements in the treatment—first, the prime necessity of limiting the quantity of food taken and the withdrawal of all proteid or xanthin-forming foods, and, secondly, with the prime importance of the care of the skin and profuse elimination from all the emunctories.

*First.*—Place the patient in bed between dry woolen blankets, wearing a light night-gown, with open sleeves fastened with safety-pins. Keep the bedclothes dry at all times and never cold and clammy; give him absolute rest and plenty of dry, warm air in a sunshiny room.

*Second.*—Withdraw all food until the alimentary canal has been thoroughly emptied and cleansed; then for the first few days employ a strictly skim-milk diet; then begin to allow stale bread and milk products. If milk is not well taken, cereal foods and buttermilk may be used. The carbonated waters often assist the difficulty in taking milk. Exclude all tea, coffee, cocoa and chocolate in the acute stages. When the patient has passed the acute stages he may gradually begin the use of fish, oysters, cottage cheese and the white meat of barnyard fowls, carefully returning to the proteid articles of diet and immediately abandoning them on a reappearance of the arthritic symptoms. Easily digested green vegetables, such as new peas, string beans, lettuce, asparagus, spinach and a small quantity of baked and mashed potatoes are allowed. Exclude all acid and sweet fruits. Oranges are freely allowed. It will be well to keep watch of excessive amounts of starchy foods and heavy sweets.

*Third.*—Give a vigorous dose of calomel, follow this with epsom or rochelle salts, and thereafter keep the bowels moving from two to four times each twenty-four hours with the salts or some other agreeable form of saline laxative. I am partial to the magnesium sulphate.

*Fourth.*—Give tub baths. Places the patient in water at 98 degrees; after he has been in the tub five minutes, add hot water gradually to bring the temperature to 100 or 102 degrees. Keep patient in the bath fifteen to twenty-five minutes, as he will bear, guarding against sensation of faintness or marked depression. Give patient skin friction, massage and passive movements entire time he is in the bath, in the order named. In removing patient from the bath be careful not to expose the skin to the air, quickly throw a dry, warm sheet and blanket around him, and get him into bed between dry, warm blankets. Now pack hot bottles about him and gradually bring on free perspiration, keeping this up from three-quarters to an hour and a half.

*Fifth.*—Now begin to give ten to fifteen grains of sodium salicylate with a glass of alkaline mineral water every two hours. Half a glass of milk may be given with a little seltzer water in place of the full glass of water if sodium salicylate causes great gastric disturbances. I seldom employ more than two drams of sodium salycilate in the twenty-four hours, and deem that dose sufficient for almost all cases. Under the treatment so far outlined one will be surprised at the size of the dose of sodium salicylate one can give without getting the customary toxic effects and gastric disturbances. Experience proves that patients taking hot baths with free elimination can take large doses of depressing drugs without the usual toxic effects, and it is very seldom that I am compelled to modify my dose of sodium salicylate. Now, as soon as the patient improves and gets comfortable, try to lengthen the interval between doses and continue the work along the line of reduction.

*Sixth.*—Flood the system by the internal administration of large quantities of an alkaline mineral water, giving it to him at regular stated intervals. If mineral water is not at hand, employ pure water, holding sodium bicarbonate or potassium citrate in solution. Keep the urine alkaline or neutral by the administration of alkaline mineral water.

*Seventh.*—Locally apply hot fomentations to the affected joints, or wrap in cotton batting or wool and apply a splint.

*Eighth.*—Unless the weather is warm, dry and free from sudden changes, keep the patient in bed for at least a week after the temperature has become normal. Caution him against exposure. It is astonishing how little exposure will bring on another attack withing a week or ten days following an apparent recovery.—*Journal A. M. A., March 3, 1906.*

---

### TWO AMAZING STORIES ABOUT LIQUOZONE.

**Editorial from *Truth*, of London, England, August 17, 1905.**

### LIQUOZONE.

With a view apparently of meeting the disclosures of a recent inquest in London, the British Liquozone Company are now distributing a new batch of testimonials to the virtues of their goods. One of these is entitled "An Opinion of Liquozone, by W. Gordon Stables, M.D., C.M. (Master in Surgery), Surgeon to the Royal Navy." Here are a few samples of W. Gordon Stables' opinion:

"Modern chemistry is every week playing into the hands of our profession, and we doctors are becoming more and more broad-minded. . . . We do not turn up our noses at every patent medicine we see on a druggist's counter, and some of these suit us admirably and come in most handy.

"I, and many hundreds of doctors know all about liquozone and what it can do, and has done, and will do. No secret is made of the formula. Its virtues are derived solely from the gases of the best producers of oxygen, such as manganese, dioxide, etc., etc. Its potent effect as a tonic is readily appreciated by medical men who have had clinical experience of its action. This is in itself enough, methinks, to give any invalid hope—a hope that is all but sure to be realized."

I do not think I can offer a better comment upon this valuable testimonial than the following extract from the *British Medical Journal* of July 29th:

"We do not know what scientific weight may attach to the investigations upon which Dr. Gordon Stables claims to express his opinion, but some of our readers may remember that in 1900 an advertising prospectus then issued by him fell into our hands, in the letter covering which, after asking for an advertisement for his forthcoming book, he said:

" 'Instead, however, of having a page advertisement at £10 10s, I should advise you to let me give you a page notice in the body of the work itself. This I will only charge £5 5s for;' while in the enclosed prospectus he stated: 'A testimonial will, as usual, be appended to each advertisement, and the doctor will make a point of frequently alluding to these in print, and in many other ways. If desired he will send an excellent brief recommendatory letter on your specialty.'

"Dr. Stables is registered as M.D., Mast. Surg., 1862, Univ.

Aberd.; he entered the Royal Navy, and was placed on the retired list on June 9, 1871. We do not think this justifies him in signing himself as 'Surgeon Royal Navy,' without adding the word 'retired.'"

To this it may be added that Dr. Gordon Stables is best known as a writer of children's books. He appears to live by literary work in this shape, eked out by supplying advertisements in the shape indicated above. He, therefore, can not be regarded as a regular medical practitioner, and it is obvious that his opinion is not of the smallest value. If it were worth anybody's while to take the matter up, it is probable that in giving this advertisement to a company of notorious quacks he would render himself liable to the forfeiture of his diploma.

Another of the new liquozone testimonials is given by a Mr. S. Rideal, of 28 Victoria street, S. W., who signs himself "D.Sc. Lond., F. I. C." Mr. Rideal certifies that after making a bacteriological examination of liquozone he found that "a 5 per cent. solution kills *B. typhosus*" in less than five minutes, whereas a corresponding solution of sulphuric acid required fifteen minutes for the same job. In a further testimonial, Mr. Rideal gives his opinion that "Liquozone is a perfectly safe preparation for adults" in the doses which he specifies. I do not understand from his degrees that he possesses any medical qualification; and his opinion as to the "safeness" of liquozone may be taken for what it is worth under those circumstances. This gentleman is, however, a doctor of science of London, and it seems a questionable proceeding for a gentleman holding that degree to supply to order certificates to a firm whose advertisements are a monument of quack imposture, and who, as Mr. Rideal can easily ascertain for himself, are holding this stuff out as a remedy for dozens of diseases which have no more relation to *B typhosus* than they have to the procession of the equinoxes.

---

Editorial from *Colllier's Weekly* for October 14, 1905.

### THE STRANGE CASE OF SMITH.

Here's a pretty story. It shows the uses to which some hospitals are put. The Suffolk Hospital, in Boston, is a so-called charity, and there are many like it. Attendance is free, but patients pay for medicine. A. C. Smith, president of the hospital and dispensary, gets free liquozone from the company and wants more. He writes and asks for the medicine in large quantities at reduced prices. He gets it. He writes enthusiastically of its merits on Suffolk paper. The superintendent of the hospital, a physician, is asked, on our behalf, by telephone, what this means. He replies that the hospital never uses liquozone. A man is sent to the hospital, also on our behalf, pretending to be a patient, and he is told also that they know nothing about liquozone. We write to the superintendent, Dr. Clarke. President Smith opens the letter and answers it, heartily indorsing liquozone, although he is not a physician and would have no right to prescribe liquozone or anything else in his hospital without the knowledge and consent of the medical staffs. We then make sure that a letter to the superintendent, Dr. Clarke, is put into his own hands, this letter telling him the whole story and asking for the explanation, to which we received no reply. Going into the matter a little further, we find a testimonial of Duffy's Malt Whiskey from A. C. Smith, on Suffolk Hospital paper, and another testimonial also written on Suffolk paper, which reads: "I can personally testify to the great remedial worth of Orangeine." It looks as if Mr. Smith's activities might diminish. Liquozone, at least, has cut him off from further supplies. How many such persons are at the head of semi-public hospitals?

---

### MELLIN'S FOOD AND THE PROPRIETARY ASSOCIATION.

**Copy of letter from Philip Mills Jones, M.D., Chairman Publication Committee of the Medical Society of the State of California, to Mellin's Food Company.**

SAN FRANCISCO, CAL., December 7, 1905.

*Mellin's Food Company of North America, Boston, Mass.*

GENTLEMEN: In view of the fact that your house deals to some extent with, and appeals for patronage to, the medical profession, and in view of the further fact that the Proprietary Association of America has organized a special bureau or committee to counteract the efforts of the medical profession to remedy the nostrum evil, and to attack its principal organization, the American Medical Association, it is with considerable surprise that we note your membership in the Proprietary Association of America, as indicated in the *Journal A. M. A.*, November 18, 1905.

We shall be pleased to receive from you some statement or explanation of your attitude toward the medical profession, so that we may present this matter fairly to the members of our Society.

We do not wish to do you or any one the slightest injustice, but we think your position rather anomalous; appealing to the medical profession for patronage, while at the same time attacking us and our American Medical Association through an association in which you hold membership, the Proprietary Association of America. Respectfully yours,

(Signed) PHILIP MILLS JONES,
Chairman Publication Committee.

Copy of letter from Mellin's Food Company to Philip Mills Jones, M.D.

BOSTON, MASS., December 29, 1905.

*Philip Mills Jones, M.D., Chairman, Publication Committee, Medical Society of the State of California, Room 1, Y. M. C. A. Building, San Francisco, Cal.*

DEAR DOCTOR: We are in receipt of your letter of December 7, 1905, making inquiry about the connection of Mellin's Food Company with the Proprietary Association of America, and asking for a statement of our attitude toward the medical profession.

We thank you for writing us in the matter, and it gives us pleasure to reply to these questions as follows:

Many years ago we joined the Proprietary Association of America, because it was simply a trade organization made up of houses whose products were sold through the same channels of trade as Mellin's Food, namely, the wholesale and retail druggists. For several years this organization continued to act as purely a trade organization, its objects being to establish such mutual cooperation as may be required in the various branches of trade; to facilitate and foster equitable principles in the purchase and sale of merchandise; to establish and maintain uniform commercial rates; and to acquire and preserve for the use of its members such business information as may prove of value to them.

Being in sympathy with these objects we continued our membership for several years, but during the past five years have not entered actively into the work of the Association.

In the present year the Association appointed a committee called the Press Committee, and the moment we learned of the plan of operations proposed by this committee we took steps to resign our membership in the Proprietary Association; and, on November 2, we mailed our resignation to the Executive Committee of the Association. Of course, the publishers of the *Journal of the American Medical Association* were not aware of this resignation when they published the list of the members of the Proprietary Association in the issue of November 18, 1905. Our resignation was accepted and we are no longer members of the Proprietary Association.

You also say in your letter: "We shall be pleased to receive from you some statement or explanation of your attitude toward the medical profession."

In reply to this we can say only that our attitude toward the medical profession always has been and is now one of profound respect, and the sincerity of our attitude is demonstrated by the action which we voluntarily took in reference to the Proprietary Association.

We may also add that the members of the medical profession have always manifested great respect for our house, and have shown entire confidence in our product. We know of no reason why this relation of mutual respect and confidence should not always continue to exist.

You will thus see that your suggestion of our attacking physicians or the American Medical Association has no foundation in fact.

We trust that you will see your way clear to present these facts to your society in accordance with the offer which you make in your letter.

With the highest regard for the Medical Society of the State of California, we beg to remain,

Very respectfully and truly yours,

MELLIN'S FOOD COMPANY OF NORTH AMERICA,

(Signed) THOMAS DOLIBER, President.

---

### CHRISTIAN SCIENCE PRACTITIONERS.

The time has passed for any especial criticism of the Christian Science cult. There have been published from time to time a number of excellent analyses of this phase of modern thought. Probably all criticism on either side are wasted time and paper, for no one is convicted to Christian Science or against it by arguments. The people who accept and practice it are as a rule impervious to any statements, no matter how strong or emphatic. They have a strain of mysticism in their compositions, and if "Mother" Eddy had not lived or wrote they would be Faith Curists, Spiritualists, Dowieites, Shakers, etc., according to the latest phase or craze. The practicing physician can console himself as to loss of practice among these people; they are rarely patients of regular practitioners.

The point which we wish to emphasize is that Christian Science is peculiarly feminine in its fascinations. Possibly it is because of "Mother" Eddy, or owing to the "father and mother God" of her prayers, or because women are more mystic in their nature. In fact, there is a strong tendency among many Christian Scientists to recognize Mother Eddy as the Shakers recognize Ann Lee, as the female embodiment of Christ. This point is futher emphasized by perusal of the principal Christian Science magazine published by the "mother" church in Boston. Everything in this religio-medical cult is "mother;" it is particularly true as the song says, "Everybody Works but Father." This paper contains the advertisements of practitioners in good standing throughout the world and may be accepted as reliable—as the cult are principally women, so the practitioners are largely women. This condition is not confined to one State, but is as true of Maine as of Arizona; in Alabama as in Idaho. Again, these practitioners are confined principally to the largest cities; thus Chicago is the hotbed of the cult, having a very large proportion of the entire number. In Alabama there are eight advertising practitioners, all women; nine live in Arkansas, all women but one. In Arizona are seven women working for the cause with not one man in the territory to charm away sorrow or error. No wonder Congress rejected this community as unfit to be a State. Science seems to be very thriving in California, if the number of practitioners is any index. There are one hundred and ninety-five women and twenty-five men practicing in that State, a large proportion being in Los Angeles. This is probably due to the fact that that town is filled with rich people more or less ill, with plenty of time on their hands. So it is in Colorado; here the thrifty Scientists are gathered together to the number of ninety-six women and seven men, busy with their little books.

Connecticut is not so fallow a field, for only twenty-eight women and five men keep up the good work here. Long experience with wooden nutmegs and other deceptive articles likely to shake faith in human nature have probably caused the people of this State to cut their eye-teeth. A curious state of affairs exists in Delaware, for there are as many men as women Scientists here, although only two of each; still Delaware is small and very busy in politics. To care for President Roosevelt and the wicked band gathered in our national capitol there are only twenty devoted women and three solitary men. Truly the task here is unequal; but it is worse in Florida, for only one man stands for the State's honor with thirteen ladies, an unlucky number.

Georgia is no better, with only two men to twenty women. Matters are also at a low ebb in Mississippi, for no men are working here and only two women. In fact Southerners do not seem to take kindly to Christian Science; probably an undue prejudice for whiskey and quinine for all ailments has crowded out the gentle theories of "Mother" Eddy.

The town of Chicago is apparently the home of Christian Science; there are nearly four hundred lady practitioners here in packing town, despite which fact we have such novels as "The

Jungle" hot from the press. New York and Philadelphia are far behind in the rate. No doubt that famous low death-rate is due to this fact; the mystery at last is solved—right under the eyes of the home of the American Medical Association do these undaunted ladies flourish, undisturbed by thoughts of ethical preparations, for to their clear vision all preparations are non-ethical. They have no star but the north star of *Science and Health,* and they sail triumphantly over the rocks and shallows that are wrecking their medical brethren. Their coin comes from an easy mint.

Idaho, again, has no male practitioner and only nine women; cowboys and miners do not seem to have illnesses of the Christian Science kind. So with the Indians—four women are enough for the Indian Territory, without male assistance of any sort except in the payment of bills. Curiously, Maryland is no more civilized; Professors Osler and Welch have no male competitors in the entire State and only a devoted band of fourteen ladies. Thus the story goes through all the States, while abroad the fad seems to have only a slight hold, if advertisements are any index.—*Medical Times, April, 1906.*

---

### ENDOMETRITIS.

By J. J. O'SULLIVAN, M.D., New York City.

Being a firm friend of glyco-thymoline for many years, I have no hesitancy in endorsing it at any time. As regards my experience with it in gynecology, will say that I have a record of some ninety cases in which I have used glyco-thymoline to a greater or less extent, and have always found it of great value in reducing congestions and engorgements and promoting a healthy condition of the tissues.

The following cases serve to illustrate the usual method followed in applying this agent:

CASE I. Mrs. H. G., aged 24; married three years; multipara; occupation, housewife; gave the following history: Began menstruating at age of 14 years, and had always been very regular during the menstrual period; had always had some pain, which had, however, become intense during the last two. Past two months had suffered backache and pain through the pelvic region; bowels constipated. Digital examination showed the cervix to be very tender and engorged, and with some slight congestion of the uterus itself, accompanied by a profuse whitish discharge. Diagnosis of endocervitis being made, a tampon of cotton soaked in pure glyco-thymoline was applied, and patient directed to inject small amount of glyco-thymoline, pure, into vagina twice a day. Tampons of cotton and glyco-thymoline were repeated every other day, and patient discharged in one month cured. Aside from an occasional saline laxative no other treatment was used.

CASE 2. Mrs. M. H., aged 21; married; multipara; occupation, housewife; Came to me complaining of intense pain throughout the pelvic region, feeling of weight and bearing-down sensation, bowels constipated and frequent micturition, having to void her urine from five to six times nightly, which was accompanied by severe burning and tenesmus. An examination disclosed a lacerated cervix with considerable inflammation of the endometrium. Treatment consisted of tampons of cotton and pure glyco-thymoline applied every second day with intrauterine douches of a hot 25% solution of glyco-thymoline applied by means of Chamberlain's glass tube. This patient has been under my treatment for three months now and the laceration has almost healed, which I expect to be complete in two to three weeks when I will discharge her, the endometrium having long since disappeared. This patient had been advised by a brother practitioner that it would be impossible to relieve the cervical laceration without an operation.

CASE 3. Mrs. McC., aged 42 years; multipara; occupation, housewife. Began menstruating at age of 13 years. Has had four children and two miscarriages. Had always experienced pain preceding periods until the last, when pain persisted throughout the week, and when I first saw her she complained of most distressing backache, bearing-down pains and pain generally throughout the entire pelvic region; associated with these symptoms was considerable vesical irritation, causing her to void urine'

four to five times every night. An examination disclosed a slight laceration of the cervix and an inflammation of the lining membrane which extended to and just beyond the internal os. Treatment consisted of tampons of cotton and glyco-thymoline, applied every third day, and hot vaginal douches of glyco-thymoline, two drachms to the pint twice a day for a week, increased to three drachms to the pint for two weeks longer, at the end of which time she was discharged cured, with directions to continue the douche for two weeks longer.

---

### SEXUAL CONTINENCE.

Can sexual continence be a cause of neurosis and psychoses? The personal qualities of the subject play an important part in this matter. Subjects in good physical and mental condition do not suffer any untoward symptoms from the practice of continence. Albrech von Heller tested on himself the effect of continence. At first he suffered from headaches and general malaise, but this was soon replaced by a condition of mental alertness and vigor. The author states that continence may be absolutely harmless when practiced by men of serious minds and wholesome mental occupations, leading regular lives and living on simple but good food. For women it is much easier to practice continence, but they should have some wholesome mental work to keep their minds busy. The degenerate and neurasthenic may find it harmful to practice continence, but sometimes they are also considerably harmed by marriage—that sometimes serves as an exciting cause of psychoses or neuroses (*Mental Diseases,* Prof. Kovalevski, pp. 232, 233, 1905).—*Journal Mental Pathology.*

---

WHEN a patient complains of dysphagia, do not neglect to examine the pericardium for effusion.—*American Journal of Surgery.*

---

IN determining the cause of a post-operative fever never fail to look at the throat.—*American Journal of Surgery.*

